# Polysulfide Concentration and Chain Length in the Biological Desulfurization Process: Effect of Biomass Concentration and the Sulfide Loading Rate

**DOI:** 10.1021/acs.est.3c03017

**Published:** 2023-08-28

**Authors:** Kestral A. K. Y. Johnston, Mark van Lankveld, Rieks de Rink, Pawel Roman, Johannes B. M. Klok, Annemerel R. Mol, Karel J. Keesman, Cees J. N. Buisman

**Affiliations:** † Environmental Technology, Wageningen University & Research, P.O. Box 17, 6700 AA Wageningen, The Netherlands; ‡ Wetsus, European Centre of Excellence for Sustainable Water Technology, Oostergoweg 9, 8911 AD Leeuwarden, The Netherlands; § Paqell B.V., Reactorweg 301, 3542 AD Utrecht, The Netherlands; ∥ Mathematical and Statistical Methods − Biometris, Wageningen University & Research, P.O. Box 16, 6700 AA Wageningen, The Netherlands

**Keywords:** biotechnology, desulfurization, polysulfides, sulfide-oxidizing-bacteria, sulfur

## Abstract

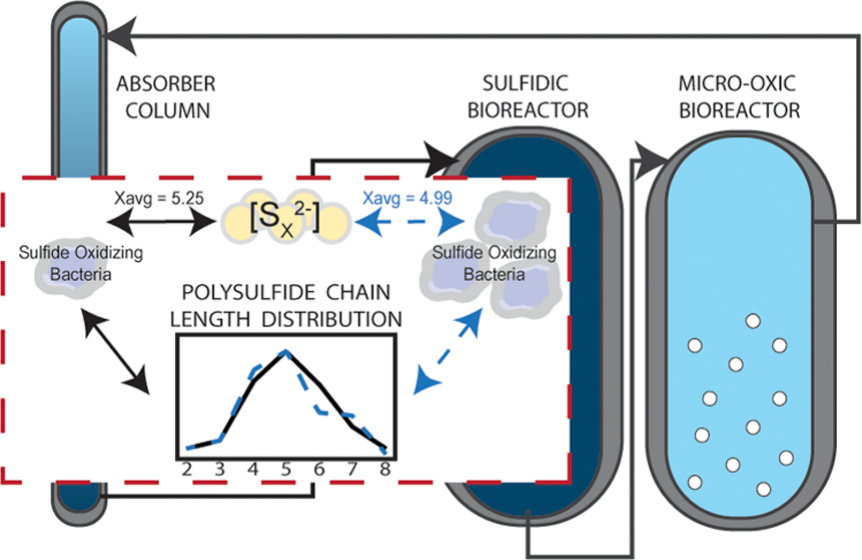

Removal of hydrogen sulfide (H_2_S) can be achieved using the sustainable biological desulfurization process, where H_2_S is converted to elemental sulfur using sulfide-oxidizing bacteria (SOB). A dual-bioreactor process was recently developed where an anaerobic (sulfidic) bioreactor was used between the absorber column and micro-oxic bioreactor. In the absorber column and sulfidic bioreactor, polysulfides (S*_x_*^2–^) are formed due to the chemical equilibrium between H_2_S and sulfur (S_8_). S*_x_*^2–^ is thought to be the intermediate for SOB to produce sulfur via H_2_S oxidation. In this study, we quantify S*_x_*^2–^, determine their chain-length distribution under high H_2_S loading rates, and elucidate the relationship between biomass and the observed biological removal of sulfides under anaerobic conditions. A linear relationship was observed between S*_x_*^2–^ concentration and H_2_S loading rates at a constant biomass concentration. Increasing biomass concentrations resulted in a lower measured S*_x_*^2–^ concentration at similar H_2_S loading rates in the sulfidic bioreactor. S*_x_*^2–^ of chain length 6 (S_6_^2–^) showed a substantial decrease at higher biomass concentrations. Identifying S*_x_*^2–^ concentrations and their chain lengths as a function of biomass concentration and the sulfide loading rate is key in understanding and controlling sulfide uptake by the SOB. This knowledge will contribute to a better understanding of how to reach and maintain a high selectivity for S_8_ formation in the dual-reactor biological desulfurization process.

## Abstract 2

Biological desulfurization of gas is a sustainable treatment technology that produces sulfur as a product. This study investigates polysulfides and their pivotal role in improving this process.

## Introduction

Biological gas desulfurization under haloalkaline conditions was developed in the 1990s^1,2^ and has been commercially applied worldwide.^3,4^ This process removes hydrogen sulfide (H_2_S) from various sour gas streams, such as natural gas and biogas, and predominantly oxidizes it to elemental sulfur (S_8_). Unlike other physical and chemical processes, the biological desulfurization process operates at ambient temperature and pressure, making it a more sustainable and cost-effective technology.^5^

The removal and conversion of H_2_S to S_8_ is achieved through a multistep process configuration that conventionally uses an absorber column and a micro-oxic bioreactor. The H_2_S-containing gas (sour gas) is fed upward through an absorber column, where it is counter-currently contacted with a (bi)carbonate process solution. H_2_S is absorbed into the haloalkaline process solution, and subsequently, the majority of the dissolved H_2_S is converted into a mixture of bisulfide (HS^–^) and polysulfides (S*_x_*^2–^). S*_x_*^2–^ is formed via the chemical equilibrium reaction between HS^–^ and S_8_ present in the solution.^6^
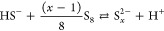
1Due to chemical equilibrium reactions, S*_x_*^2–^ of chain length *x* exists in solution and is distributed over the range of 2–9. This mechanism can be generally expressed via the following chemical reaction (eq 2).^7^
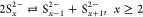
2The process solution containing (poly)sulfides leaves the absorber column and is fed to a micro-oxic bioreactor where sulfide-oxidizing bacteria (SOB) convert the (poly)sulfides in solution to S_8_, forming a solid fraction.^8^ The end-product S_8_ can be removed from the process via, for example, centrifugation. After removal and dewatering, S_8_ can be used in agricultural applications.^9^

In the biological desulfurization process, sulfate (SO_4_^2–^) and thiosulfate (S_2_O_3_^2–^) are produced as unwanted acidifying by-products. Sulfate is produced via the biological oxidation of HS^–^ and S_2_O_3_^2–^,^10^ and S_2_O_3_^2–^ is produced through the chemical oxidation of HS^–^ and S*_x_*^2–^.^11^ The formation of both by-products has been shown to be highly influenced by the operational process parameters.^12−14^ By-product formation results in the need for additional caustic and makeup water, a bleed stream, and greater usage of oxygen and nutrients, all of which increase operational costs.

In recent years, a new process configuration has been introduced that uses an anaerobic (sulfidic) bioreactor, i.e., nonaerated and at high sulfide concentrations, located in the flow scheme between the absorber column and the micro-oxic bioreactor.^15^ This dual-bioreactor process scheme has been proven to decrease the formation of both SO_4_^2–^ and S_2_O_3_^2–^. The formation for SO_4_^2–^ decreased because the sulfidic conditions in the sulfidic bioreactor inhibited a key enzymatic pathway. The formation for S_2_O_3_^2–^ decreased because part of (poly)sulfide was removed from the solution in the sulfidic bioreactor, resulting in a lower concentration entering the micro-oxic bioreactor.^15,16^

In addition to limiting by-product formation, the new biological desulfurization configuration has also provided insight into the abilities of the SOB. It has been observed that the SOB possesses an electron shuttling capacity, i.e., the bacteria catalyze the redox reactions and act as both an electron acceptor and an electron donor. SOB can partially remove (poly)sulfide in the sulfidic bioreactor and later reduce oxygen within the micro-oxic bioreactor.^17^ The main biological and chemical reactions of (poly)sulfide removal, especially within the solution, have been studied, and recent experiments have found the removal to be more efficient at higher pH and higher sulfide loads.^16,18^ However, the underlying mechanisms of (poly)sulfide removal within and near the SOB remain to be determined.

**Figure 1:**
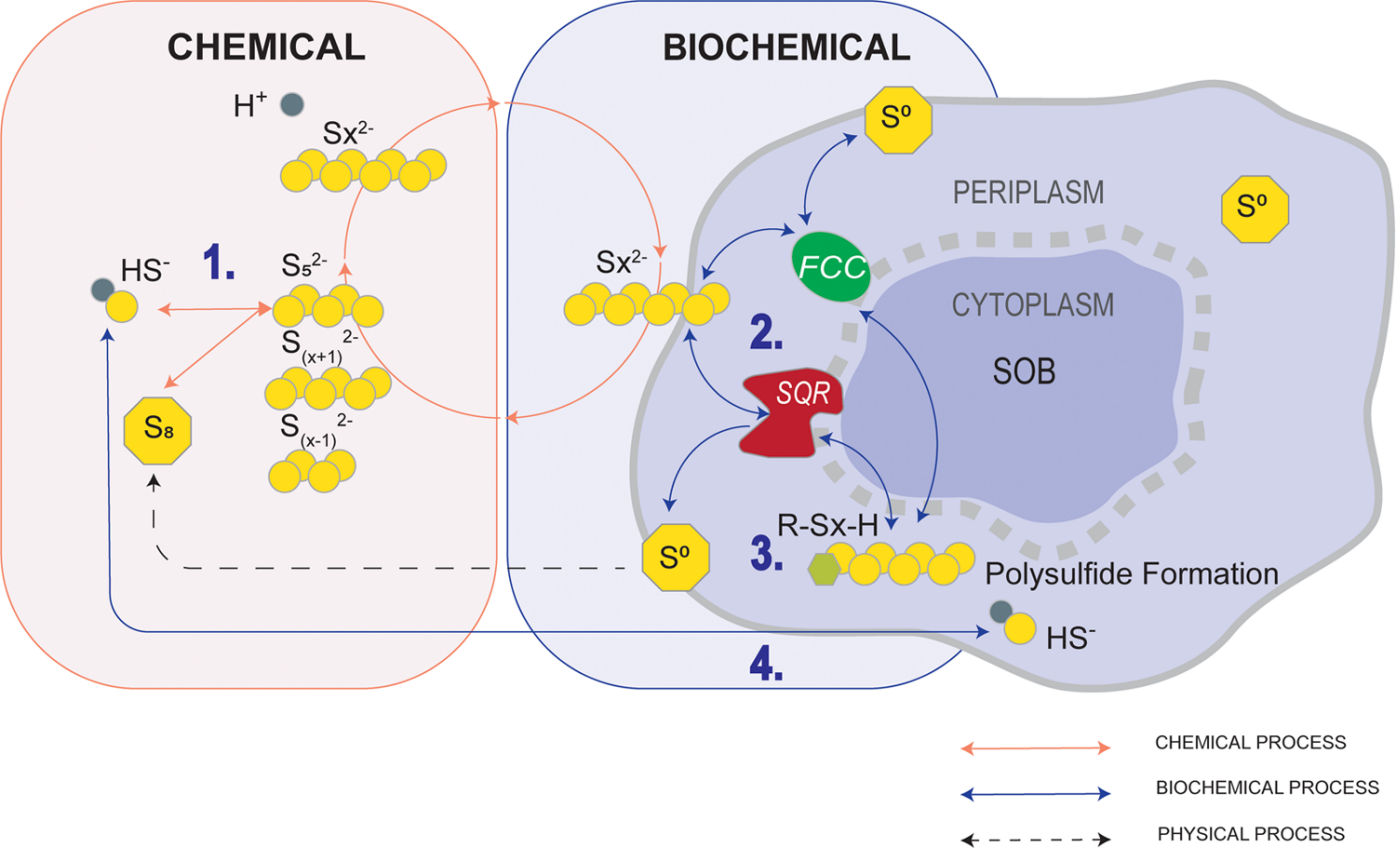
Summary of the current knowledge on the multiple pathways between chemical S*_x_*^2–^ formation outside of the cell and the biochemical formation and breakdown of S*_x_*^2–^ in and around the sulfide-oxidizing bacteria (SOB). Illustrated above, (1) chemically, sulfide and sulfur react in solution to form S*_x_*^2–^. In the biochemical processes, current knowledge illustrates (2) the potential use of S*_x_*^2–^ by the enzyme system SQR or FCC,^20,26,27^ followed by (3) the formation of sulfur and potentially S*_x_*^2–^;^28^ (4) sulfide is known to be able to cross-cell membranes.^29^

Previous research has determined that SOB can remove S*_x_*^2–^ in solution and biologically oxidize it to elemental sulfur.^19,20^ S*_x_*^2–^ can potentially be consumed and/or produced by the enzymes within the SOB (Figure 1). Within the SOB, sulfide oxidation to elemental sulfur can be performed by either flavocytochrome sulfide dehydrogenase (FCC) or sulfide:quinone reductase (SQR).^21,22^ The FCC route is shown to be suppressed when exposed to high sulfide concentrations leading SQR to become dominant.^10,15,23^ Previous studies have shown that SQR produces soluble S*_x_*^2–^ as the primary product^24^ and that S*_x_*^2–^ can be present in the cells of SOB^25^ or used as the intermediate in the production of sulfate.^20,26^ Therefore, SOB exposed to (poly)sulfides may be able to store and further oxidize them to elemental sulfur.

In addition to biological interactions, chemical S*_x_*^2–^ equilibrium and distribution have been studied extensively without biology.^6,30,31^ Calculated thermodynamic constants (p*K*) are reported between 9.18 and 14.43 for S*_x_*^2–^ of chain lengths 2–8,^31^ and for biologically produced sulfur, a p*K* value of 9.17 has been reported.^31,32^ In addition to p*K* values, the concentration of S*_x_*^2–^ has been shown to increase with pH in excess of HS^–^ and S^0^, the distribution of S*_x_*^2–^ does not change from pH of ∼7 to 12, and equilibrium between S*_x_*^2–^ species occurs rapidly in the order of 10 s.^33^ However, the rate at which equilibrium is reached depends on HS^–^ concentration, pH, temperature, and the state of elemental sulfur, making measurements necessary to understand S*_x_*^2–^ in solution.^34−36^

S*_x_*^2–^ concentrations and their chain lengths were previously measured by Roman et al. within a full-scale biological desulfurization process at the outlet of the absorber column, but with the single reactor configuration. Additionally, the samples were stabilized after 2 h of transportation time, which permitted (bio)chemical reactions to take place. In follow-up studies, S*_x_*^2–^ concentrations were measured from samples that were stabilized immediately in controlled lab-scale sulfidic bioreactors. These experiments, however, focused on the removal of thiols and were performed under low volumetric H_2_S loading rates typically not seen in practice.^37,38^ Considering these factors, S*_x_*^2–^ has yet to be measured under controlled conditions and at sulfide concentrations like those seen in industrial applications. Therefore, the role of all and specific species of S*_x_*^2–^ in the biological desulfurization process remains unknown despite it being pivotal to understanding how to design the sulfidic bioreactor for the best S_8_ selectivity. Improving the S_8_ selectivity can improve the efficiency and stability of the biological desulfurization process, which can increase its implementation over other desulfurization technologies.

The aim of this study was to assess S*_x_*^2–^ concentrations and their chain lengths under industrially relevant conditions utilizing a pilot-scale biological desulfurization setup with a sulfidic bioreactor. Additionally, the study aimed to elucidate a relationship between S*_x_*^2–^ concentrations and chain length and the biomass concentration in the sulfidic bioreactor.

## Materials and Methods

### Experimental Setup

**Figure 2:**
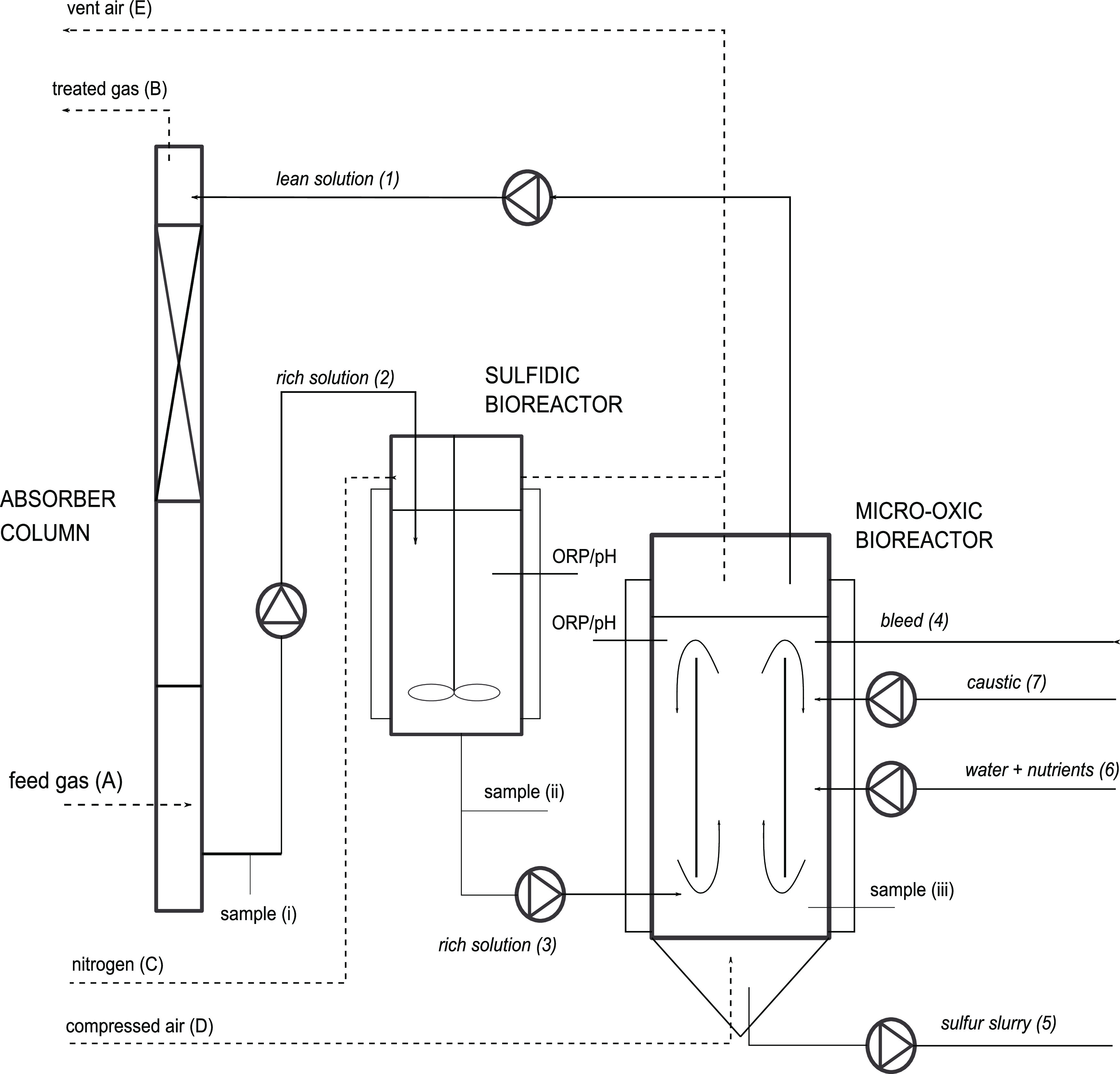
Schematic overview of the pilot system with the gas (dashed line) and liquid (solid line) flows. More details of the setup can be found elsewhere.^15^

The experimental setup used a pilot-scale biological desulfurization installation, which included a pressurized absorber column maintained between 2.7 and 3.0 bar (g), sulfidic bioreactor, micro-oxic bioreactor, and decanter centrifuge (Figure 2). A mixture of nitrogen (N_2_), carbon dioxide (CO_2_), and H_2_S gases entered below the packing material in the absorber column. Each gas was supplied and controlled separately through mass flow controllers (Profibus, Brooks instruments, Hatfield, PA). The sump of the absorber column (where the sulfidic solution was collected) had a total liquid volume of 1.0 L. The liquid volumes were set at 2.4 and 11.4 L in the sulfidic bioreactor and micro-oxic bioreactor, respectively. The sulfidic bioreactor was equipped with a mechanical mixer (rzr2020, Heidolph Instruments, Schwabach, Germany), and N_2_ was injected into the headspace to ensure anaerobic conditions. Both bioreactors were equipped with water jackets connected to a thermostat bath (Kobold, Germany) to keep the bioreactor liquid temperatures constant at ∼35 °C.

Pumps continuously circulated the process liquid containing buffered medium, sulfur particles, dissolved sulfur species, and SOB over the entire system. The solution leaving the absorber column contained dissolved sulfide (i.e., H_2_S, HS^–^, S*_x_*^2–^, and S^2–^ “rich” solution), whereas dissolved sulfide could not be detected in the solution entering the absorber column from the micro-oxic bioreactor (“lean” solution). Nutrients, caustic solution, and makeup water (∼150 mL day^–1^) were continuously supplied to the bioreactor. The same nutrient solution used by de Rink et al. was supplied for the growth of the bacteria. Caustic was dosed to maintain a constant alkalinity of 0.5 M HCO_3_^–^, and makeup water was supplied to maintain a constant conductivity (i.e., salinity) of 55 mS-cm^–1^. Solution left the system at an average flow of 1.2 kg day^–1^ over 3 months through an overflow from the micro-oxic bioreactor, giving the system a total hydraulic retention time (HRT) of ∼14 days.

During the experiments, the oxidation–reduction potential (ORP) and pH were continuously measured with a probe (SE552/2 Inducon ORP/pH sensor) connected to a Stratos Pro Transmitter (Knick, Berlin, Germany). An integrated Ag/AgCl electrode was a reference for both ORP and pH.

### Experimental Operation

The bioreactor was inoculated with a bleed solution from the dual-reactor pilot unit that had been stored at 4 °C. The total experiment duration was one month, in which the H_2_S loading rate and biomass concentration were varied. During this period, the composition of the SOB microbial community was monitored weekly using next-generation sequencing (NGS). Detailed sample preparation, analyses, and bioinformatics information can be found in Supporting Information 1.

The setup was continuously operated with 10 kg h^–1^ of liquid circulating throughout the system. At this circulation rate, the HRT was approximately 5 min for the absorber column, 15 min for the sulfidic bioreactor, and 45 min for the micro-oxic bioreactor. The pressure in the absorber column was kept between 2.7 and 3.0 bar (g). A constant stream of 100 Ln h^–1^ (normal liters) of N_2_ flowed to the absorber column. The CO_2_ flow varied between 15 and 40 Ln h^–1^ as it fluctuated to maintain the system’s pH. The pH of the micro-oxic bioreactor was between 8.3 and 8.7, with an average of 8.48 ± 0.10, while the pH of the sulfidic bioreactor varied between 7.7 and 8.0. The solution’s alkalinity was, on average, 0.54 M ± 0.12 M (total concentration of NaHCO_3_ and Na_2_CO_3_ and expressed as the concentration of Na^+^). The H_2_S flow varied between 0.8 and 4.4 Ln h^–1^, corresponding to ∼30 to 150 g-S day^–1^. The lowest H_2_S flow rate had a volumetric loading rate of 1.92 g-S L^–1^day^–1^, more than 3 times higher than previous volumetric H_2_S loads in lab-scale bioreactors.^37,38^ Compressed air was supplied to the micro-oxic bioreactor with the flow rate controlled by a PI controller to maintain the ORP value set-point at −350 mV, with the average being −349 ± 21 mV throughout the experiment.

Liquid samples were taken from the absorber column and the sulfidic bioreactor. The first sampling port was located at the bottom of the absorber column before the sulfidic bioreactor, and the second sampling port was located in a recirculation line of the sulfidic bioreactor (Figure 2). The H_2_S load was increased incrementally (∼10 g-S day^–1^ each time) over the duration of multiple weeks. Samples were taken at H_2_S loading rates of 27, 38, 47, and 58 g-S day^–1^ over the entire system volume of 14.3 L at two different biomass concentrations, 24 and 90 mg-N L^–1^ to study the influence of biomass on the S*_x_*^2–^ concentration and chain-length distribution. Full-scale units typically operate at biomass concentrations between 50 and 100 mg-N L^–1^. Therefore, 24 mg-N L^–1^ is considered “low” as it is half of the typical biomass concentration.^15^ To achieve the higher biomass concentration, the nutrient dosing rate was increased in the micro-oxic bioreactor.

Changing the H_2_S inflow to the absorber caused fluctuations in the ORP of the micro-oxic bioreactor. Therefore, samples were taken only after a minimum 1 h wait time—15 min for the ORP to stabilize again plus a minimum of 45 min (3 HRTs) for the sulfidic bioreactor to reach a steady state.

### Batch Bottle Control Experiments

Abiotic batch bottle experiments were conducted to determine the S*_x_*^2–^ concentration and distribution under abiotic conditions. Batch bottles were chosen, as continuous operation of the pilot desulfurization system is unfeasible without the presence of SOB, as hydrogen sulfide would build up over time. During the experiment, only sulfide, biologically produced sulfur, and carbonate medium were added to the batch bottles. Biologically produced sulfur was washed with Milli-Q water and centrifuged 3 times. With each centrifuging step, biomass was scraped from the surface of the pellet and removed. The resulting biosulfur was analyzed using a scanning electron microscope (SEM) to ensure biomass was no longer attached. Sodium carbonate medium was created using 77 g-L^–1^ of NaHCO_3_, 4.8 g-L^–1^ of Na_2_CO_3_, 1 g-L^–1^ of K_2_HPO_4_, and 0.2 g-L^–1^ of MgCl_2_·6H_2_O. The resulting solution had a Na^+^ concentration of 1 M and a pH of ∼8.5. Micronutrients were not added to the medium, as trace metals are known to have a catalytic effect on S*_x_*^2–^.

A 100 mM-S sulfide stock solution was made using NaHS·9H_2_O. This solution was added in different volumes to create initial sulfide concentrations similar to those found in the pilot with 3.9, 5.2, 6.5, and 7.9 mM-S initial sulfide concentrations and 4.7, 6.3, 7.9, and 9.4 mM-S sulfur concentrations. Sulfur was always in excess, with 20% more sulfur than the highest sulfide concentration.

The bottles were left to mix overnight on a shake table at 25 °C. Samples were taken from the bottles the next day within an anoxic environment, and the same analysis method was used to determine S*_x_*^2–^ and their chain lengths. Sample analysis was performed in triplicate. All sample volume was taken from the same sampling syringe.

### Analyses

#### Biomass Analysis

Biomass concentration was quantified spectrophotometrically based on the total amount of organic nitrogen (N) oxidized to nitrate by ammonium persulfate (LCK138, Hach Lange, Tiel, The Netherlands). Biomass-N is an indicator of the total biomass in the system, which includes SOB based on NGS data (Supporting Information 1). A sample was taken from the micro-oxic bioreactor and split into two separate parts. One part was centrifuged for 10 min at 15 000 rpm, while the other part was left uncentrifuged. The total concentration of biomass in terms of nitrogen was determined by subtracting the supernatant total N from the total N of the uncentrifuged samples. Biologically produced sulfur has been found to not interfere with the results if the samples were 5 times diluted.^14,15^ The haloalkaline SOB has the generic stoichiometric chemical equation CH_1.8_O_0.5_N_0.2_ where N is 10% mole of the total dry-weight biomass, allowing for indirect characterization of the biomass.^20^ Since the biological desulfurization system is a continuous process in a steady state, it can be assumed that biomass concentrations are uniform throughout the system.

#### Sample Preparation for Polysulfide Analysis

S*_x_*^2–^ samples were stabilized by using the procedure described in Roman et al.^37^ (Supporting Information 2). The pH for each sample was determined by using a pH probe prior to stabilizing the samples with methyl triflate (≥98% pure, Sigma-Aldrich, The Netherlands) to form more stable dimethyl polysulfanes (eq 3).^7^
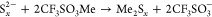
3After methylation and the addition of the internal standard (dibenzo-a, h-anthracene, Supelco Analytical) dissolved in benzene (Sigma-Aldrich, The Netherlands), samples were stored at 4 °C for no more than 4 days. Samples were centrifuged at 3300*g* for 10 min to ensure any remaining particles had settled out of the solution before analysis.

#### Polysulfide Analysis

The samples were analyzed using an ultra-high-performance liquid chromatograph (uHPLC) with a UV detector (Dionex UtliMate 3000RS) to determine the separate fractions of different chain lengths of S*_x_*^2–^ in solution. The uHPLC was equipped with an Agilent column (Zorbax Extend-C18 18 μm, 2.1 × 50 mm^2^) operated at 20 °C, and the UV detector was set to 210 nm. For the uHPLC analysis, the flow rate was 0.371 mL min^–1^ with 1.25 μL injection volume. First, a mobile phase consisting of a methanol (15% vol) and water (85% by vol) mixture entered the column. After 0.72 min, a convex gradient developed until methanol reached 85% vol after 10 min had passed. For the next 10 min, the system was isocratic. In the following 5 min, methanol decreased to 15% vol. The last 5 min of the uHPLC run were isocratic again.

The concentration of individual S*_x_*^2–^ chain lengths (2–8 sulfur atoms) was determined using the peak areas from the uHPLC column, internal standard, and response factors (RF) from Roman et al. Total S*_x_*^2–^ concentration is the sum of all of the chain lengths, and the average chain length was calculated based on the S*_x_*^2–^ fraction of each chain length present in the samples. All samples were analyzed in at least triplicate from the same sampling syringe.

## Results and Discussion

### Total Polysulfide Concentration Varies With Increased Sulfide Loading Rates

The pilot-scale biological desulfurization system was operated for 24 days during which the sulfide loading rate was incrementally increased from 28 to 151 g-S day^–1^. S*_x_*^2–^ concentrations and their chain lengths (*x* = 2–8) were determined in the rich solution in the absorber column sump and the sulfidic bioreactor.

**Figure 3:**
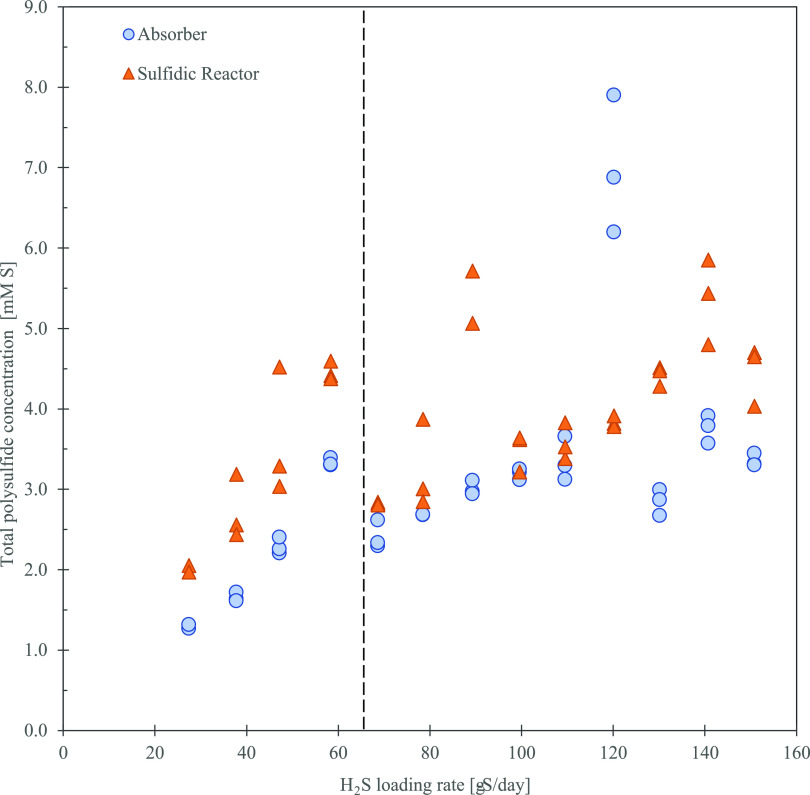
Total S*_x_*^2–^ concentration (mM-S) at increasing sulfide loading rates (g-S day^–1^) in the rich solution of the absorber column and sulfidic bioreactor. The dashed vertical line indicates when the nutrient dosing was increased to grow more biomass to handle the increased H_2_S supply to the system.

The total S*_x_*^2–^ concentration ranged from 1.3 to 7.9 mM-S in the absorber column and 2.0–5.9 mM-S in the sulfidic bioreactor (Figure 3). Excluding one loading rate (121 g-S day^–1^), the sulfidic bioreactor contained, on average, 45% more S*_x_*^2–^ than the absorber column. A strong linear correlation was observed between total S*_x_*^2–^ concentration and H_2_S loading rates of 28–69 g-S day^–1^. However, total S*_x_*^2–^ concentration varied once the H_2_S loading rate increased beyond 69 g-S day^–1^ (Figure 3). Other operational process parameters, such as the pH and ORP, remained constant throughout the entire experiment. The most likely explanation for this abrupt decrease in total S*_x_*^2–^ concentration would be a change in biomass concentration as more bacteria enabled higher cell membrane permeation of S*_x_*^2–^ into the cells.^29,39^ Over the course of the experiment, the biomass concentration increased 68% from ∼24 to 90 mg-N L^–1^ (Supporting Information 3).

Even though the chemical conditions are similar for both solutions in the absorber column and sulfidic bioreactor, a difference in S*_x_*^2–^ concentration was observed. The average concentrations of S*_x_*^2–^ were found to be in the range of 2.69 ± 1.10 mM-S (with the outlier at 15.7 mM-S removed) in the absorber column and 3.80 ± 1.17 mM-S in the sulfidic bioreactor (Figure 3). The difference in total S*_x_*^2–^ concentration was expected due to the difference in HRTs between the two reactor sections (i.e., 5 min for the absorber column and 20 min in total (5 + 15) for the sulfidic bioreactor), which may not be sufficient time to reach equilibrium, as described in a later section.

**Figure 4:**
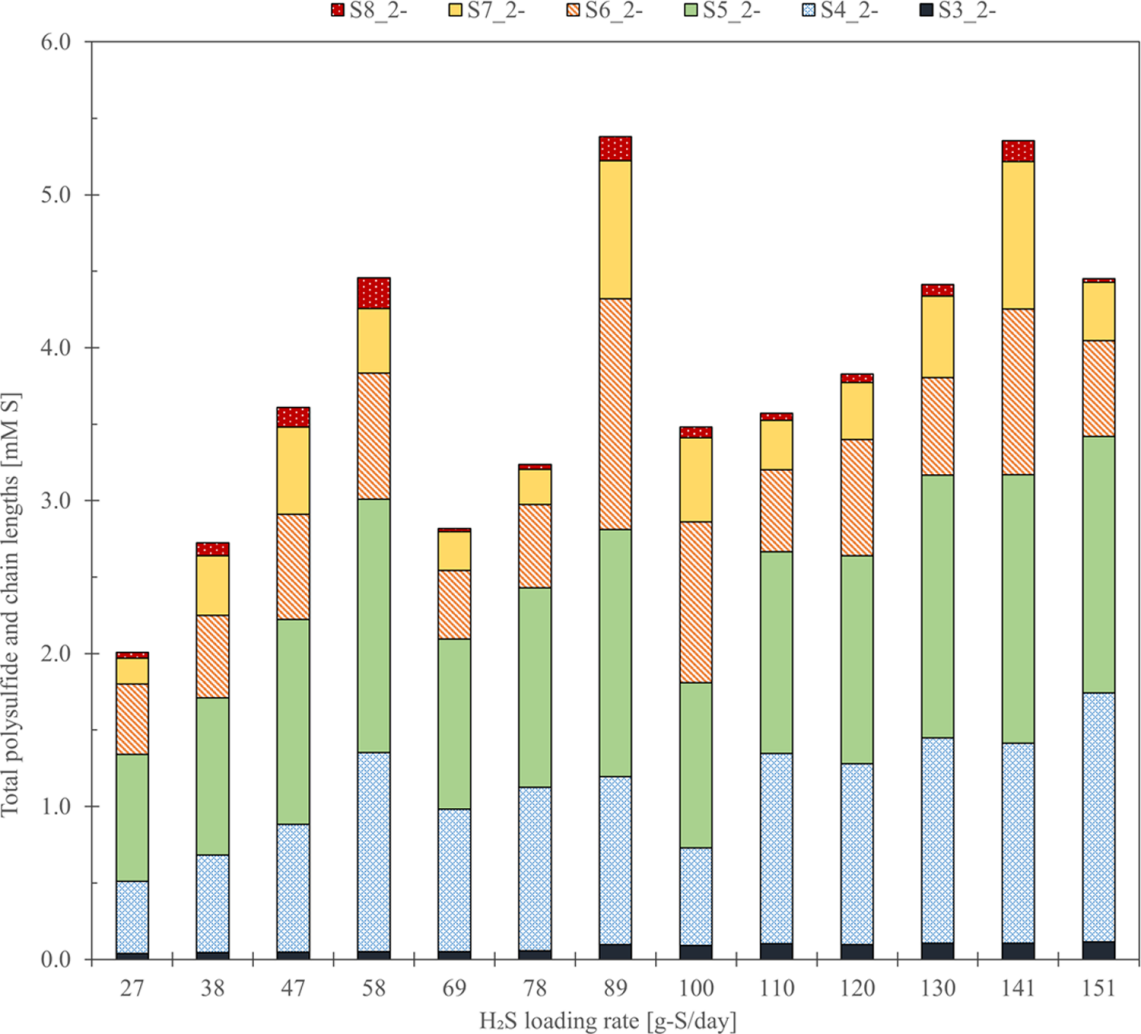
Distribution of S*_x_*^2–^ chain lengths was determined over increasing sulfide loading rates. S_2_^2–^ was present at values below 0.3% and was excluded from the graph as its values were negligible in comparison with the other chain-length percentages (more than a factor of 10 lower than the next lowest chain length). Raw data can be found in Supporting Information 4.

In addition to the total S*_x_*^2–^ concentration, individual S*_x_*^2–^ anions were determined. Results showed that pentasulfide (S_5_^2–^) was the predominant species for all conditions, which is in agreement with previous results^37^ (see also eqs 4 and 5). The formation of S_5_^2–^ leads to the formation of tetrasulfide (S_4_^2–^), hexasulfide (S_6_^2–^), trisulfide (S_3_^2–^), heptasulfide (S_7_^2–^), disulfide (S_2_^2–^), and octasulfide (S_8_^2–^) (Figure 4).
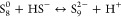
4
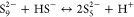
5

During this experiment, S_8_^2–^ was present at all loading rates even though S_8_^2–^ had not been detected in prior experiments using either lab setups or a full-scale installation.^37,38^ The absence of S_8_^2–^ in previous experiments can most likely be attributed to three reasons: (1) lower sulfide concentrations and sulfide:sulfur ratios, (2) the time between sampling and sample derivatization, and (3) the sulfidic retention time. First, previous lab-scale experiments were conducted at much lower sulfide levels (between 0.16 and 0.24 mM-S), leading to lower overall S*_x_*^2–^ concentrations (eq 1).^37,38^ Greater concentrations of S*_x_*^2–^ in solution can lead to larger chain lengths, such as S_8_^2–^ being present at quantifiable concentrations. Second, immediate stabilization was not possible for S*_x_*^2–^ from the prior full-scale installation experiments. Before S*_x_*^2–^ was derivatized, samples had to be transported to the laboratory, which took around 2 h. Therefore, the chemical equilibrium in the solution likely changed due to the presence of active biomass and potential oxidation. Finally, S*_x_*^2–^ formation is highly influenced by the sulfidic retention time, which was limited in both previous studies due to experimental setup constraints.

### Impact of Biomass Concentration on the Concentration and Chain-Length of Individual Polysulfide Anions in the Process Solution

After the initial experiment where only the H_2_S loading on the system increased, the first four H_2_S loading rates (27, 38, 47, and 58 g-S day^–1^) were repeated using a higher biomass concentration. The amount of biomass increased from 24 to 90 mg-N L^–1^ by increasing the nutrient dosing rate.

**Figure 5:**
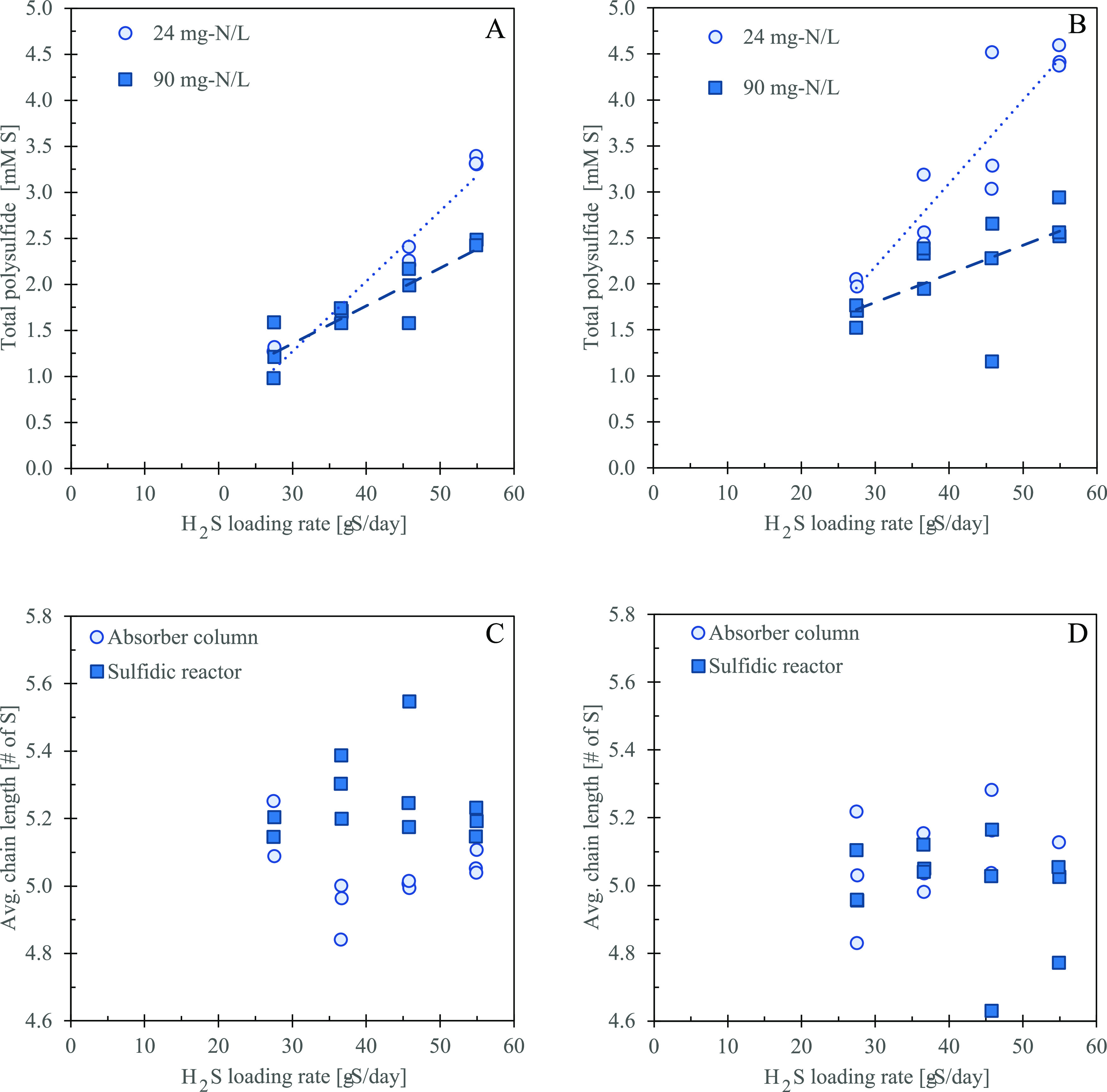
Total S*_x_*^2–^ within the system at a pH set-point of 8.5 versus the H_2_S loading rate under low (24 mg-N L^–1^) and high (90 mg-N L^–1^) biomass concentrations in both the (A) absorber column and (B) sulfidic bioreactor. The dashed lines on the graph are placed as a guide for the eye. The average chain length of S*_x_*^2–^ was determined within the system at a pH set-point of 8.5 versus the concentration of sulfide within the absorber column and sulfidic bioreactor during the operations with (C) low (24 mg-N L^–1^) and (D) high (90 mg-N L^–1^) biomass concentrations.

At the initial H_2_S loading rate of 27 g-S day^–1^ (theoretical concentration of 3.93 mM-S of sulfide), the total S*_x_*^2–^ concentration in the absorber was 1.29 ± 0.04 mM-S at low biomass and 1.25 ± 0.10 mM-S at high biomass, while the total S*_x_*^2–^ concentration in the sulfidic bioreactor was 2.01 ± 0.06 mM-S at low biomass and 1.67 ± 0.07 mM-S at high biomass. These starting values are similar to each other despite the fact that biomass concentration increased by a factor of ∼4. Once the dissolved sulfide concentration was increased, total S*_x_*^2–^ increased for both biomass concentrations and in both the absorber column and the sulfidic bioreactor (Figure 5A,B). The total S*_x_*^2–^ concentration was lower at high biomass concentrations (90 mg-N L^–1^) than at low biomass concentrations (24 mg-N L^–1^). The greatest difference in total S*_x_*^2–^ concentration between the low and high biomass concentrations was at the loading rate of 58 g-S day^–1^ (7.85 mM-S of sulfide). At the 58 g-S day^–1^ loading rate, a difference of 0.88 ± 0.08 mM-S in the absorber and 1.79 ± 0.14 mM-S in the sulfidic bioreactor was observed.

As previously mentioned, the differences between the absorber column and sulfidic bioreactor can most likely be attributed to different HRTs. The absorber column had a 5 min HRT, whereas the sulfidic bioreactor had a 15 min HRT on top of the 5 min already spent in the absorber column. This lower total S*_x_*^2–^ concentration in the absorber suggests that HS^–^ and S_8_ had not yet reached equilibrium with S*_x_*^2–^ in the absorber column. Previous studies show that the rate at which chemical S*_x_*^2–^ equilibrium occurs is dependent on process conditions such as HS^–^ concentration, pH, temperature, and the state of the elemental sulfur.^34−36^ Since the conditions in the system were similar, most likely, the SOB influenced the equilibrium of S*_x_*^2–^ in solution since the concentration of biomass increased by a factor of almost 4. Thus, similar observations in the absorber column are made for low and high biomass, as the HRT was not long enough for the SOB to influence the concentration of total S*_x_*^2–^.

To further understand the results obtained in the pilot experiment, batch bottles were used to obtain data on S*_x_*^2–^ in solution without biomass. When the solution is undersaturated with respect to sulfur, overnight mixing is sufficient for chemical equilibrium to be reached.^40^ If, however, the solution is supersaturated with regards to sulfur, the kinetics will change and equilibrium may take much longer.^41^ Total S*_x_*^2–^ concentrations resulted in a linear relationship with a resulting slope similar to that from the data obtained from the lower biomass concentration experiment, but with a smaller intercept (1 mM-S at sulfide concentration of 4 mM-S). This similar slope in the line indicates that experiments with a low biomass concentration and with no biomass have similar equilibria in solution. With the addition of more biomass, there is a shift in equilibrium, as shown by the decrease in slope. It is hypothesized that as the ratio of sulfide to biomass is higher, the chemical reactions dominate the solution, as sulfur is always in excess.

When the biomass concentration was increased, the total biomass (which included the SOB) was in excess and able to grow to a concentration that is unachievable at the low H_2_S loading rates. Our hypothesis is that the excessive SOB caused a shift in S*_x_*^2–^ equilibrium by increasing the amount of SOB–S*_x_*^2–^ interactions. Biologically, S*_x_*^2–^ can cross over the cell membrane due to their lipophilicity and can interact with the sulfur-producing enzymes within the SOB.^29,42^ For example, the SOB with the SQR enzyme has been shown to store S*_x_*^2–^ in their periplasm in the form of organic S*_x_*^2–^ and make less S*_x_*^2–^ available in solution to form more S*_x_*^2–^.^43^ With an excessive amount of biomass, more S*_x_*^2–^ could be stored within the SOB because their main form of substrate is limited, forcing them into “starvation” and increasing storage. Thus, less S*_x_*^2–^ could be present in the bulk solution as these SOB are no longer “fully active” due to the lack of a substrate.

The average chain length for both high and low biomass operations was determined by calculating the weighted average of S*_x_*^2–^ with 2–8 sulfur atoms (Figure 5C,D). At low biomass, the average chain length for the four H_2_S loading rates was 5.05 ± 0.02 for the absorber and 5.25 ± 0.03 for the sulfidic bioreactor. At higher biomass levels, the average chain length for the four sulfide loading rates was 5.08 ± 0.04 for the absorber and 4.99 ± 0.06 for the sulfidic bioreactor. When comparing the measurements from the absorber column and the sulfidic bioreactor, S*_x_*^2–^ in the absorber column were, on average, 0.2 sulfur atoms shorter than in the sulfidic bioreactor and had a lower average chain length at 75% of the time (Figure 5C). As for the higher biomass concentration, no distinct difference in the S*_x_*^2–^ chain length between the absorber column and sulfidic bioreactor could be observed (Figure 5D).

Since the system operational parameters remained constant throughout the experiment, it is presumed that the equilibrium of the bulk solution did not significantly impact the average S*_x_*^2–^ chain length at the excess biomass concentration. We hypothesize that the higher average S*_x_*^2–^ chain length in the sulfidic bioreactor compared to the absorber column at the lower biomass concentration could be explained due to the limited 5 min HRT of the absorber column. As the system is assumed to be at a steady state, the SOB could shift the system equilibrium if they are at a high enough concentration and utilize S_x_^2–^ as a substrate. At a lower biomass concentration, we hypothesize that this “fully active” biomass could assist in the production of longer S*_x_*^2–^ chains. Previous research has shown S*_x_*^2–^ to be microbiologically produced, and could be excreted by the SOB or react with sulfur globules inside the cell.^25,33^

**Figure 6:**
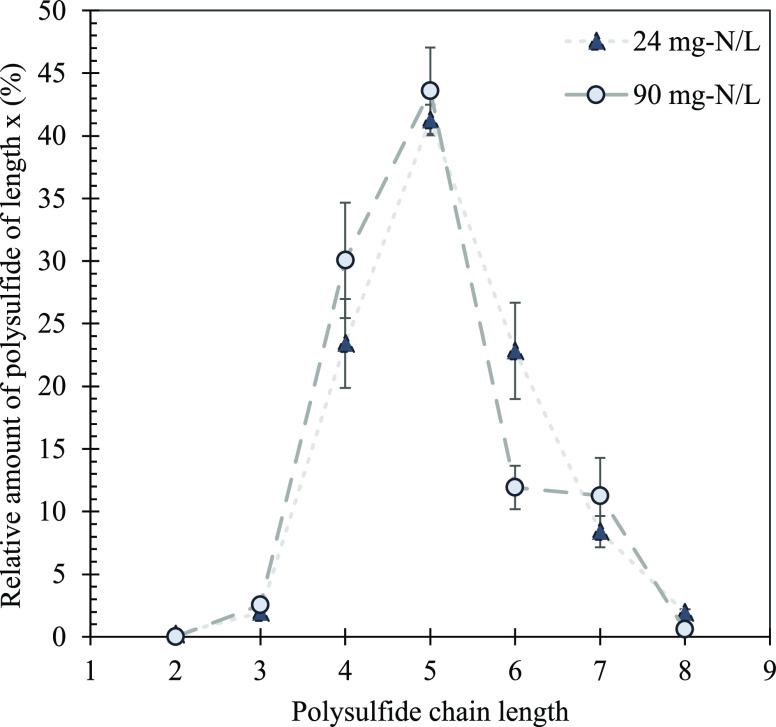
Profile of the distribution of S*_x_*^2–^ chain lengths for the 27 g-S day^–1^ loading rate in the sulfidic bioreactor for low and high biomass concentrations.

In addition to the average chain length, the chain-length distribution was compared between both the low and high biomass concentrations (Figure 6). With no biology present, a distribution based on eq 2 forms. Pentasulfide (S_5_^2–^) is the first to form and continues to form mixtures of S*_x_*^2–^ ions, leading to the formation of other chain lengths.^37^ A close to the normal distribution is expected based on this chemical equilibrium with S_5_^2–^ as the most dominant chain length. At the lower biomass concentration (i.e., 24 mg-N L^–1^), S_5_^2–^ is the dominant chain length with the other chain lengths, following the “normal” distribution pattern. However, at the higher biomass concentration (90 mg-N L^–1^), a different distribution is seen with S_5_^2–^ still being the most prevalent chain length, but an increase in S_4_^2–^ and S_7_^2–^ and a substantial decrease in S_6_^2–^ can be observed. This pattern occurs in both the absorber column and sulfidic bioreactor for all of the repeated sulfide loading rates (Supporting Information 5).

During the high biomass experiment, the SOB could be considered “in excess,” making the biochemical reactions that utilize S*_x_*^2–^ dominant in comparison to the chemical reactions. Therefore, the distribution at high biomass was disturbed due to the excess amount of SOB in solution, causing S*_x_*^2–^ with chain lengths of 6 and 8 to decrease, while chain lengths of 4 and 7 increased. We hypothesize that the SOB influences S*_x_*^2–^ through one or only a few chain lengths to disrupt the typical distribution. Even though S*_x_*^2–^ is lipophilic, potentially, the charge over the entire S*_x_*^2–^ molecule influences what can pass through the periplasmic membrane of the SOB, i.e., the larger the chain length, the less relative charge the entire S*_x_*^2–^ molecule has. Additionally, the enzymes within the SOB could prefer certain chain lengths over others due to adapted binding sites, suggesting a preference for S_6_^2–^ and S_8_^2–^. However, more research is needed to understand the complex relationship between the SOB and S*_x_*^2–^ of specific chain lengths.

## Considerations

In this study, S*_x_*^2–^ of chain lengths between 3 and 8 were detected and quantified in the biological desulfurization process. Up until this work, S_8_^2–^ had not been detected in the biological desulfurization system, as previous work only found chain lengths of 2–7. The concentration of S*_x_*^2–^ in the absorber column was consistently less than the concentration in the sulfidic bioreactor. This difference in concentration can be attributed to the difference in HRTs between the absorber column (5 min) and sulfidic bioreactor (5 + 15 min).

The biological desulfurization system is complex due to the multiple physical and chemical properties that can affect S*_x_*^2–^ concentration and chain length. Therefore, to elucidate the relationship between S*_x_*^2–^, H_2_S loading rate, and biomass, only the H_2_S loading rates and biomass concentration were varied. This study found that the presence and concentration of biomass influence the concentration of S*_x_*^2–^ in solution and the chain-length distribution. When the biomass concentration increased, the total concentration of S*_x_*^2–^ decreased at the four different H_2_S loading rates. The lower biomass concentration resulted in a difference in the average S*_x_*^2–^ chain length between the absorber column and sulfidic bioreactor, where the average S*_x_*^2–^ chain length was lower for the absorber column. We hypothesize that the difference in chain lengths was most likely due to longer chains of S*_x_*^2–^ being taken up by the SOB or that the SOB facilitates the production of S*_x_*^2–^ when given a longer time exposed to sulfidic conditions. Additionally, changes in relative concentration were observed between S*_x_*^2–^ chain lengths where S_6_^2–^ was found to decrease, while S_4_^2–^ and S_7_^2–^ increased at high biomass concentrations. More research is needed to determine if the SOB prefers certain chain lengths and if intracellular S*_x_*^2–^ accumulates. However, it seems apparent that the SOB uses S*_x_*^2–^ as an intermediate in the sulfidic bioreactor, which is a major step forward in understanding reaction mechanisms in the biological desulfurization process.

## Supporting Information Available

(1) Detailed NGS materials, methods, and results; (2) detailed information on the polysulfide sample preparation; (3) biomass concentration results during the experiment; (4) detailed polysulfide chain-length distribution results; (5) summary of average polysulfide chain lengths results, and (6) results of chain-length profiles of additional H_2_S loading rates (PDF)
