# Supplementary material for: Polysulfide Concentration and Chain Length in the Biological Desulfurization Process: Effect of Biomass Concentration and the Sulfide Loading Rate
Source: Environ Sci Technol. 2023 Aug 28;57(36):13530–40. doi: 10.1021/acs.est.3c03017 (PMC10501124; doi:10.1021/acs.est.3c03017)
Supplement: Supplementary file 1 — es3c03017_si_001.pdf [file es3c03017_si_001.pdf]

Supporting Information

Polysulfide concentration and chain length in the biological desulfurization process: Effect of biomass concentration and sulfide loading rate

Kestral A.K.Y. Johnston,[1,2] Mark van Lankveld,[1,3] Rieks de Rink,[1,3] Pawel Roman,[2] Johannes B.M. Klok,[2] Annemerel R. Mol,[*,1] Karel J. Keesman,[2,4] Cees J.N. Buisman [1,2]

1. Environmental Technology, Wageningen University & Research, P.O. Box 17, 6700 AA, Wageningen, The Netherlands
2. Wetsus, European Centre of Excellence for Sustainable Water Technology, Oostergoweg 9, 8911 AD Leeuwarden, The Netherlands
3. Paqell B.V., Reactorweg 301, 3542 AD Utrecht, The Netherlands
4. Mathematical and Statistical Methods – Biometris, Wageningen University & Research, P.O. Box 16, 6700 AA, Wageningen, The Netherlands

*Corresponding author: Annemerel R. Mol – Phone: +31(0)317483339; Email: annemerel.mol@wur.nl

Summary:

The supporting information for the manuscript "Polysulfide concentration and chain length in the biodesulfurization process: Effect of biomass concentration and sulfide loading rate" contains 12 pages, consisting of 8 figures, and 1 table.

Contents:

SI 1 – NGS data for starting biomass and throughout the experiment

SI 2 – Detailed information on polysulfide sample preparation

SI 3 – Biomass concentration data while changing H_2S loading rate

SI 4 – Data polysulfide distribution with error

SI 5 – Summary of average polysulfide chain lengths

SI 6 – Chain length profiles for H_2S loading rates (27, 38, 47, and 58 gS/day)

References

SI 1 – NGS data for starting biomass and throughout the experiment

Materials and Methods

The microbial community in the pilot biological desulfurization system was monitored using 16S rRNA gene amplicon sequencing. Duplicate samples of 2 mL were collected weekly throughout the experiment from the micro-oxic bioreactor. Samples were centrifuged in Eppendorf tubes for 10 min at 15,000 rpm. Afterwards, the supernatant was removed, and the sample was immediately snap-frozen in liquid nitrogen. Before DNA extraction, samples were kept at -80°C. DNA extraction was performed using the DNeasy PowerLyzer PowerSoil Kit (Qiagen) according to the manufacturer's instructions. DNA purity and concentration was measured using a NanoDrop® spectrophotometer (Thermo Fisher Scientific, Germany). DNA extracts were normalized to 20 ng/µL and sent for library preparation and 16S rRNA gene amplicon sequencing on an Illumina MiSeq at MrDNAlab (Shallowater, TX, USA). The bioinformatics was performed according to [1], except that taxonomic classification was based on SILVA v.132 [2].

Raw sequence data was deposited in the European Nucleotide Archive (ENA) project PRJEB64350. The bioinformatics scripts (QIIME2 files) are available upon request.

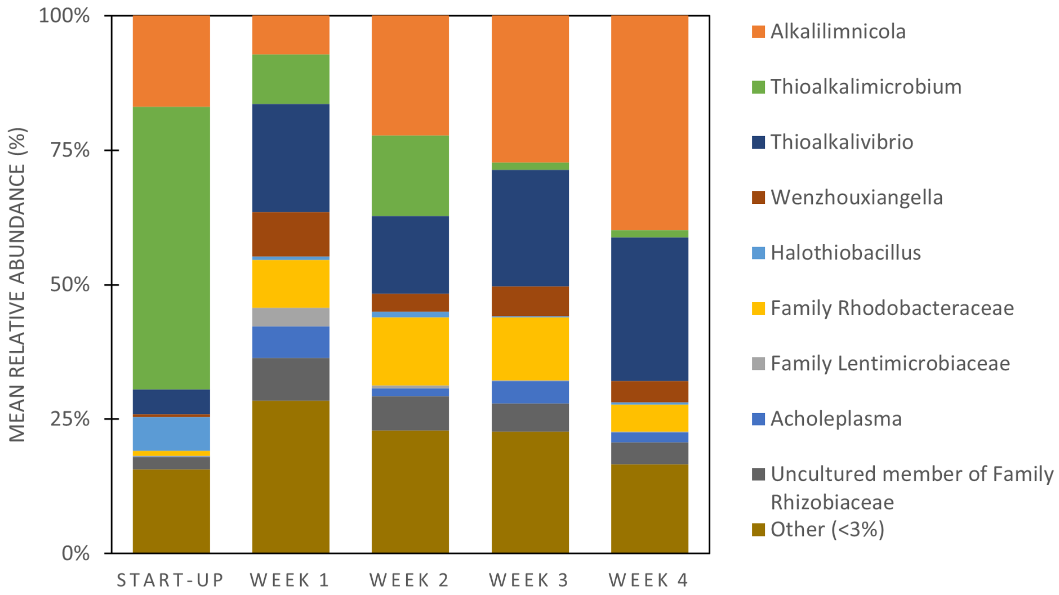

Figure SI 1 – Relative abundance of microbial groups (Genus level) throughout the experiment measured by 16S rRNA gene amplicon sequencing (NGS).

Based on the relative abundances seen in Figure SI 1, genera *Alkalilimnicola, Thioalkalimicrobium, and Thioalkalivibrio* were continuously present throughout the experiment and made up ~30-60% of the microbial community. The observed shifts in the microbial community during the experiment are hypothesized to be due to the increased H_2S loading rate. The increase of *Alkalilimnicola* over time due to high H_2S loading rates is in accordance with previous research [3,4].

SI 2 – Detailed information on polysulfide sample preparation

Before taking a sample, each port was flushed three times with a glass syringe. The syringe was used to take the sample and immediately transferred to an anaerobic tent. All samples were derivized with methyl triflate as quickly as possible, i.e. within a matter of minutes. No significant difference was found between samples that were methylated between the initial sample time and two hours after sampling. The amount of methyl triflate (MTF) added to each sample was determined according to Kamyshny et al., 2006 as the pH influences the amount of MTF needed to completely derivatize the polysulfides but also not to overestimate their concentration in solution.

While in the anaerobic tent, samples were first passed through a 0.7 µm glass fiber filter (AP40, 25 mm, Millipore, USA) encased in a metal holder (Microsyringe Filter Holder 25 mm, Merck). After filtering, samples were kept in glass vials and capped in the anaerobic chamber. To ensure polysulfides did not adsorb onto any plastic surfaces, the sample was shaken with extra care to ensure that the liquid did not touch the cap. To prepare each sample, 800 µL of methanol (flushed with nitrogen gas for a minimum of 20 min) was added to a glass 2 mL vial. After the methanol addition, 200 µL of sample and the calculated amount of methyl triflate were added simultaneously to the vial. The sample was taken with a glass syringe while the methyl triflate was pipetted using a 200 µL pipette. The vial was sealed and rotated three times to ensure mixing. The vial was reopened, and 20 µL of an internal standard (dibenzo-a, h-anthracene, Supelco Analytical, USA) dissolved in benzene (Sigma-Aldrich, Netherlands) was added.

SI 3 – Biomass concentration data while changing H_2S loading rate

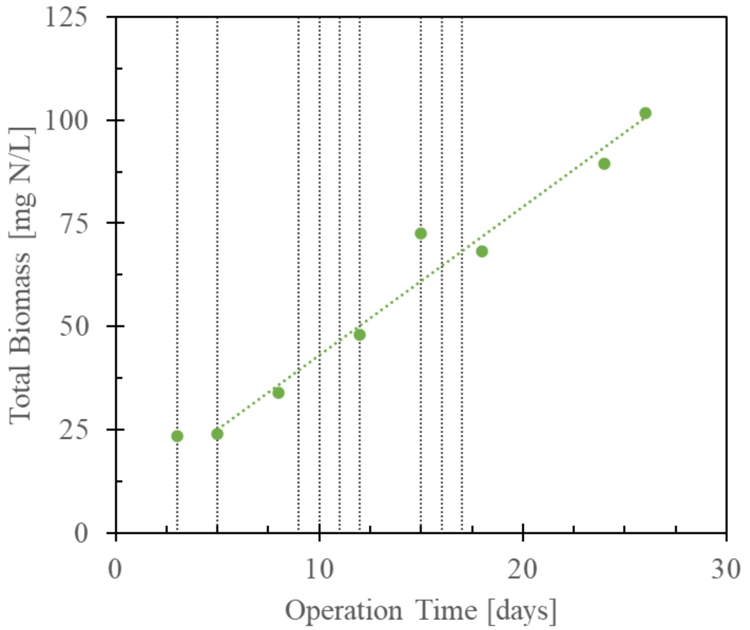

Figure SI 3.1 – Biomass concentrations versus polysulfide sampling. Vertical dashed lines indicate polysulfide sampling points.

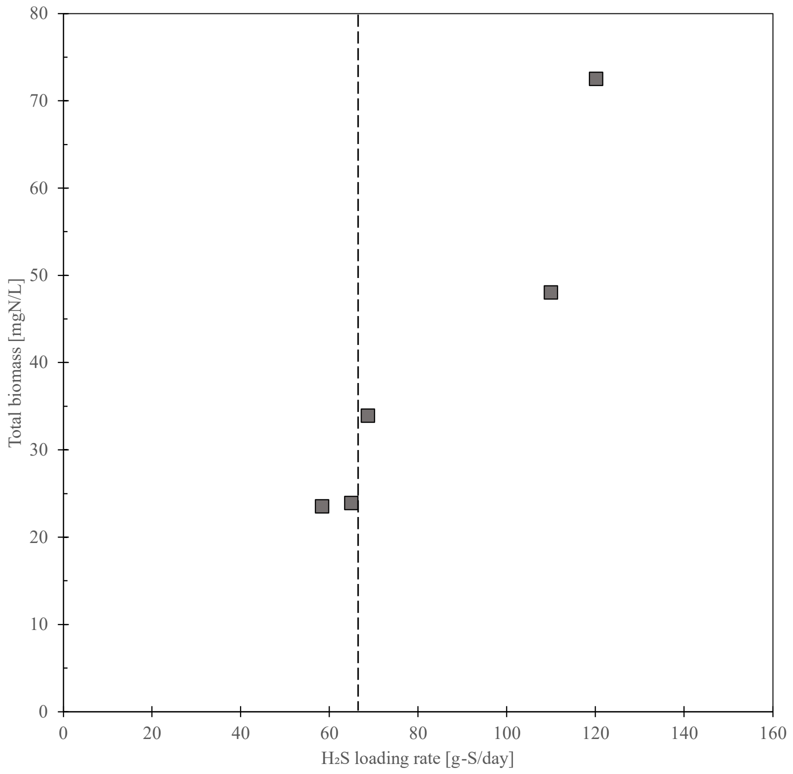

Figure SI 3.2 – Comparison with biomass concentrations at certain H2S loading rates – vertical line indicates when nutrient solution dosing rate was increased for biomass growth

SI 4 – Data polysulfide distribution with error

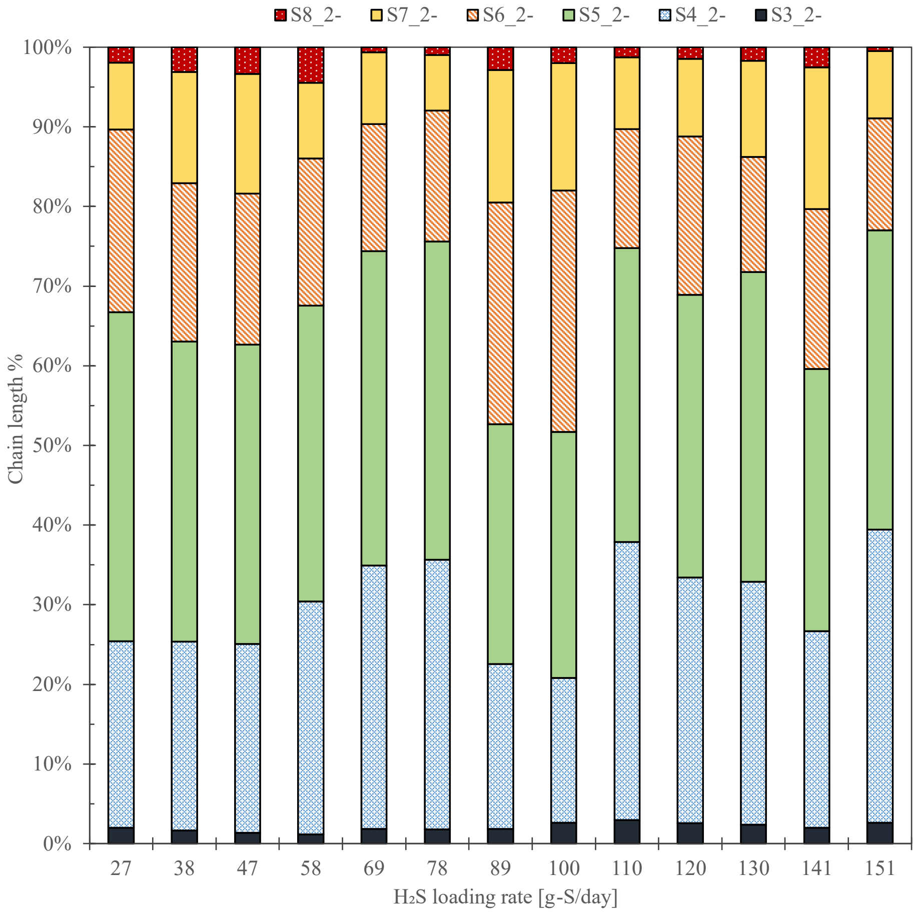

Figure SI 4.1 – Distribution of S_x^{2-} chain lengths based on normalization of S_x^{2-} concentrations and raw data with error deviations

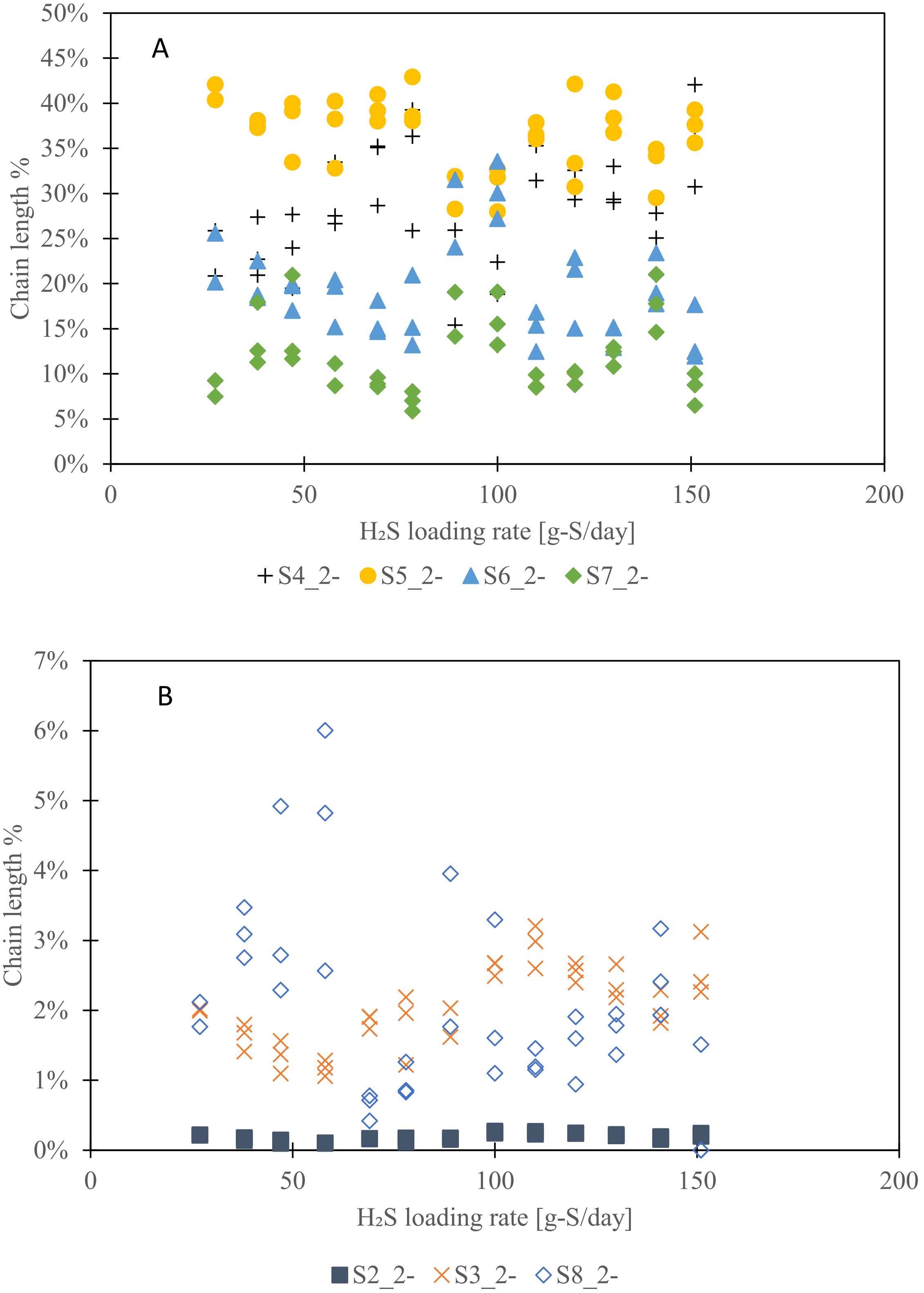

Figure SI 4.2 – Distribution of S_x^{2-} chain lengths based on percentage of total S_x^{2-} including all data points (triplicates) A) S_x^{2-} where x is between and including 4 – 7 B) S_x^{2-} where x is 2, 3 and 8

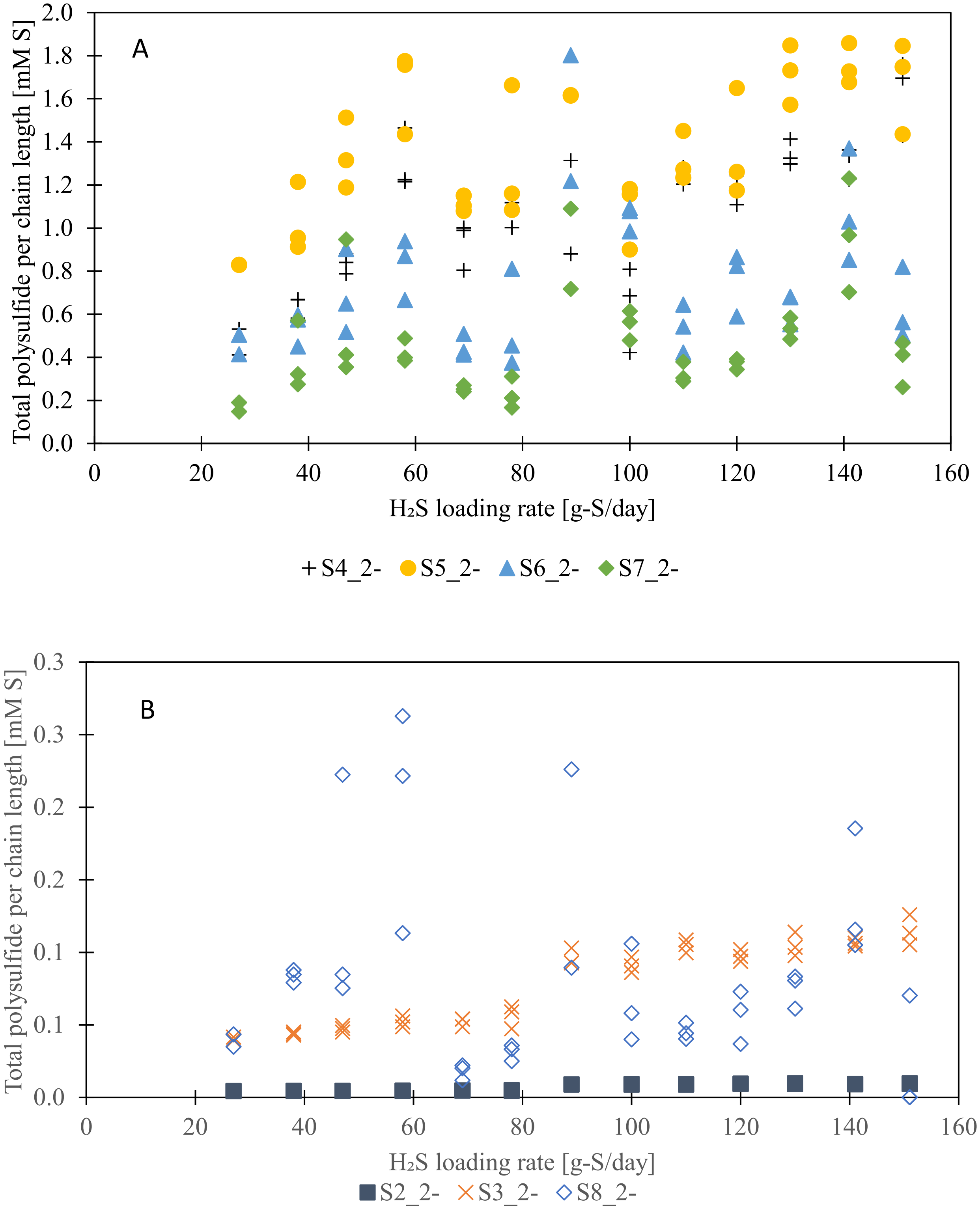

Figure SI 4.3 – Total S_x^{2-} concentration of each chain length including all data points (triplicates) A) S_x^{2-} where x is between and including 4 – 7 B) S_x^{2-} where x is 2, 3 and 8

SI 5 – Summary of average polysulfide chain lengths

Table SI 5. Summary of average chain lengths within the biological desulfurization system at different biomass concentrations.

	mM of S	Absorber column	Sulfidic reactor
**Low biomass (24 mgN/L)**	3.9	5.17 ± 0.04	5.18 ± 0.06
	5.2	4.94 ± 0.05	5.30 ± 0.05
	6.5	5.01 ± 0.05	5.32 ± 0.08
	7.9	5.07 ± 0.04	5.19 ± 0.06
**High biomass (90 mgN/L)**	3.9	5.03 ± 0.10	5.01 ± 0.07
	5.2	5.06 ± 0.07	5.07 ± 0.03
	6.5	5.16 ± 0.07	4.94 ± 0.22
	7.9	5.08 ± 0.04	4.95 ± 0.07

SI 6 - Chain length profiles for H_2S loading rates (27, 38, 47, and 58 gS/day)

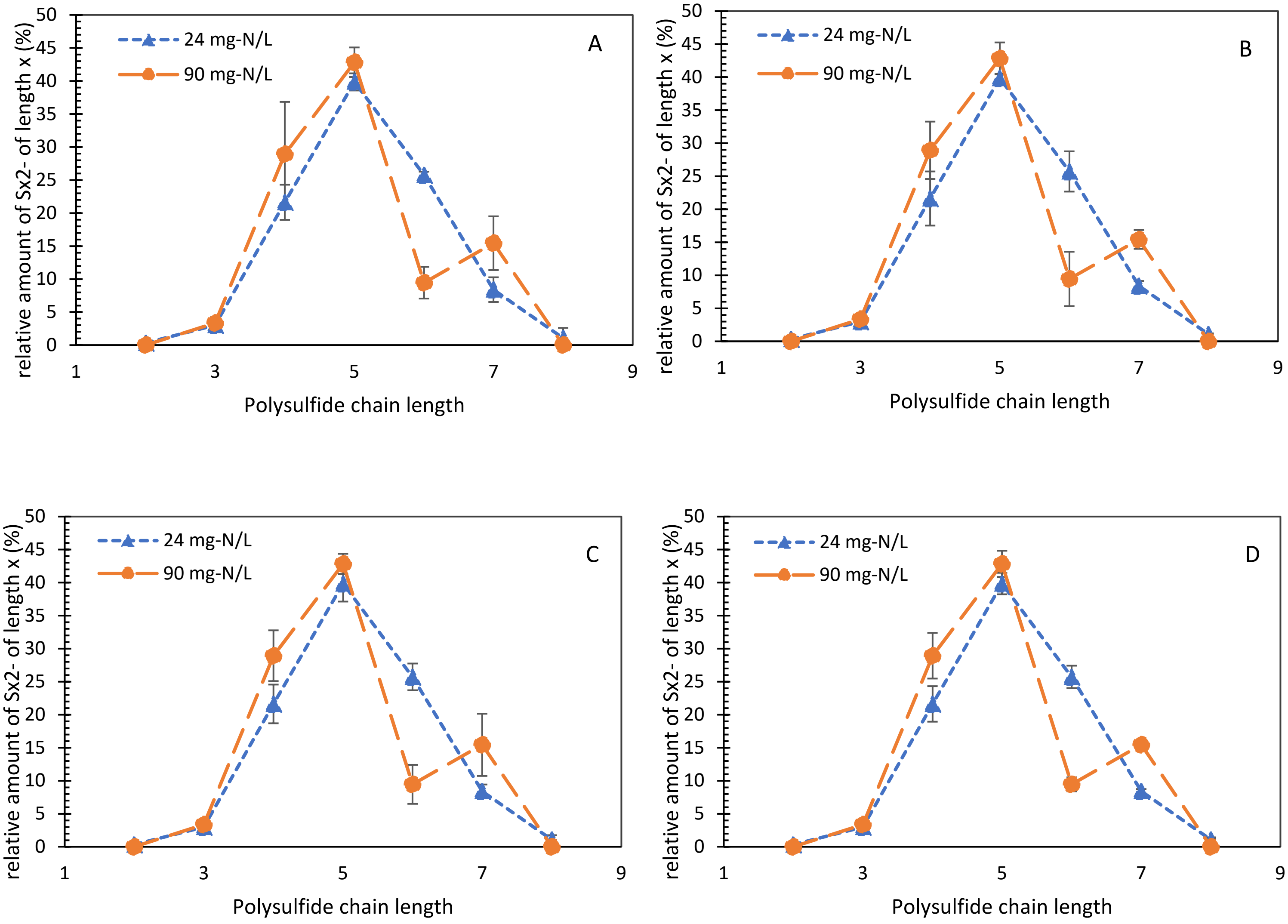

Figure SI 6.1 – Chain length profiles for H_2S loading rates A) 27, B) 38, C) 47, and D) 58 g-S/day for the absorber column at low and high biomass concentrations

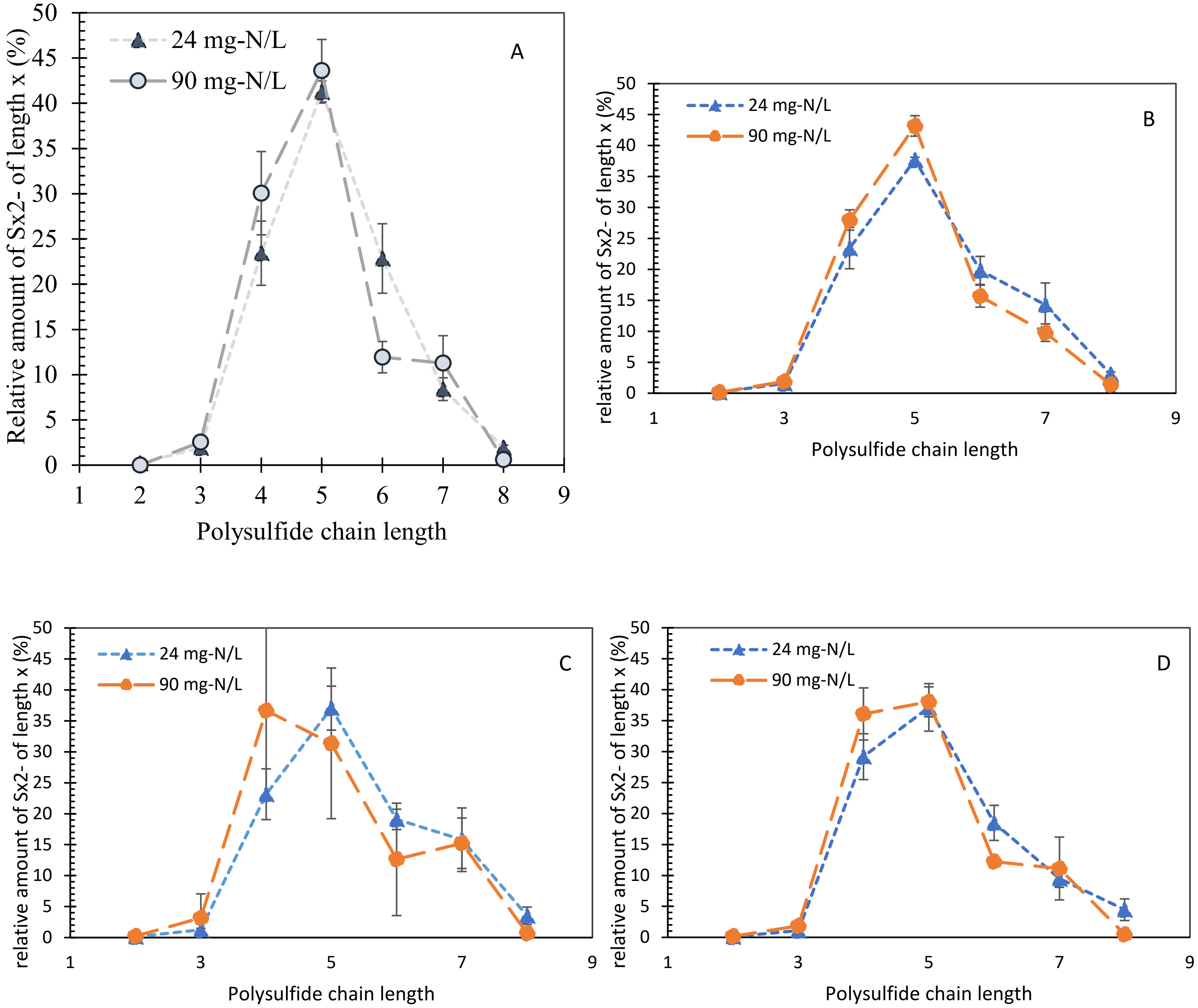

Figure SI 6.2 – Chain length profiles for H_2S loading rates H_2S loading rates A) 27, B) 38, C) 47, and D) 58 g-S/day for the sulfidic bioreactor at low and high biomass concentrations

References

(1) Estévez-Alonso, Á.; Arias-Buendía, M.; Pei, R.; van Veelen, H. P. J.; van Loosdrecht, M. C. M.; Kleerebezem, R.; Werker, A. Calcium Enhances Polyhydroxyalkanoate Production and Promotes Selective Growth of the Polyhydroxyalkanoate-Storing Biomass in Municipal Activated Sludge. *Water Res* **2022**, *226*. https://doi.org/10.1016/j.watres.2022.119259.

(2) Quast, C.; Pruesse, E.; Yilmaz, P.; Gerken, J.; Schweer, T.; Yarza, P.; Peplies, J.; Glöckner, F. O. The SILVA Ribosomal RNA Gene Database Project: Improved Data Processing and Web-Based Tools. *Nucleic Acids Res* **2013**, *41* (Database issue), D590-6. https://doi.org/10.1093/nar/gks1219.

(3) De Rink, R.; Klok, J. B. M.; Van Heeringen, G. J.; Sorokin, D. Y.; Ter Heijne, A.; Zeijlmaker, R.; Mos, Y. M.; De Wilde, V.; Keesman, K. J.; Buisman, C. J. N. Increasing the Selectivity for Sulfur Formation in Biological Gas Desulfurization. *Environ Sci Technol* **2019**, *53* (8), 4519–4527. https://doi.org/10.1021/acs.est.8b06749.

(4) de Rink, R.; Gupta, S.; Piccioli de Carolis, F.; Liu, D.; ter Heijne, A.; Klok, J. B. M.; Buisman, C. J. N. Effect of Process Conditions on the Performance of a Dual-Reactor Biodesulfurization Process. *J Environ Chem Eng* **2021**, *9* (6), 106450. https://doi.org/10.1016/j.jece.2021.106450.

(5) Kamyshny, A.; Ekeltchik, I.; Gun, J.; Lev, O. Method for the Determination of Inorganic Polysulfide Distribution in Aquatic Systems. *Anal Chem* **2006**, *78* (8), 2631–2639. https://doi.org/10.1021/ac051854a.
